# Persistent symptoms are diverse and associated with health concerns and impaired quality of life in patients with paediatric coeliac disease diagnosis after transition to adulthood

**DOI:** 10.1136/bmjgast-2022-000914

**Published:** 2022-07-12

**Authors:** Satu Vuolle, Pilvi Laurikka, Marleena Repo, Heini Huhtala, Katri Kaukinen, Kalle Kurppa, Laura Kivelä

**Affiliations:** 1 Celiac Disease Research Center, Faculty of Medicine and Health Technology, Tampere University, Tampere, Finland; 2 Tampere Centre for Child, Adolescent and Maternal Health Research, Faculty of Medicine and Health Technology, Tampere University, Tampere, Finland; 3 Department of Pediatrics, Tampere University Hospital, Tampere, Finland; 4 Department of Internal Medicine, Tampere University Hospital, Tampere, Finland; 5 Department of Pediatrics, Central Finland Central Hospital, Jyväskylä, Finland; 6 Faculty of Social Sciences, Tampere University, Tampere, Finland; 7 The University Consortium of Seinäjoki, Seinäjoki, Finland; 8 Children's Hospital, and Pediatric Research Center, University of Helsinki and Helsinki University Hospital, Helsinki, Finland

**Keywords:** COELIAC DISEASE, GLUTEN FREE DIET, PAEDIATRIC GASTROENTEROLOGY

## Abstract

**Objective:**

To investigate the prevalence and associated factors of persistent symptoms despite a strict gluten-free diet in adult patients with coeliac disease diagnosed in childhood.

**Design:**

Medical data on 239 currently adult patients with paediatric diagnosis were collected from patient records. Also, patients completed structured study questionnaire. All variables were compared between those with and without persistent symptoms.

**Results:**

Altogether 180 patients reported adhering to a strict gluten-free diet. Of these, 18% experienced persistent symptoms, including various gastrointestinal symptoms (73%), arthralgia (39%), fatigue (39%), skin symptoms (12%) and depression (6%). Those reporting persistent symptoms had more often gastrointestinal comorbidities (19% vs 6%, p=0.023), health concerns (30% vs 12%, p=0.006) and experiences of restrictions on daily life (64% vs 43%, p=0.028) than the asymptomatic subjects. The patients with symptoms had poorer general health (median score 13 vs 14, p=0.040) and vitality (15 vs 18, p=0.015) based on a validated Psychological General Well-Being Questionnaire and more severe symptoms on a Gastrointestinal Symptom Rating Scale scale (total score 2.1 vs 1.7, p<0.001). Except for general health, these differences remained significant after adjusting for comorbidities. The groups were comparable in current sociodemographic characteristics. Furthermore, none of the childhood features, including clinical, serological and histological presentation at diagnosis, and adherence and response to the diet after 6–24 months predicted symptom persistence in adulthood.

**Conclusion:**

Almost one-fifth of adult patients diagnosed in childhood reported persistent symptoms despite a strict gluten-free diet. The ongoing symptoms were associated with health concerns and impaired quality of life.

**What is already known on this topic**

- The most common reason for persistent symptoms in coeliac disease is non-adherence to a gluten-free diet.
- Some patients nevertheless suffer from persistent symptoms despite successful dietary treatment.
- Prevalence and associated factors of long-term persistent symptoms in patients diagnosed in childhood and adhering to a strict diet are unclear.

**What this study adds**

- Almost one-fifth of currently adult patients diagnosed in childhood with coeliac disease report persistent symptoms despite a strict gluten-free diet.
- Patients report both gastrointestinal symptoms and a variety of extraintestinal complaints.
- Persistent symptoms may impair self-perceived quality of life and cause unnecessary health concerns.

**How this study might affect research, practice and/or policy**

- Efforts should be made to organise optimal support and evaluations to rule out possible comorbidities in patients with coeliac disease suffering from persistent symptoms.

## Introduction

Coeliac disease is a gluten-driven gastrointestinal disorder affecting approximately 1% of the population.^1 2^ Its clinical presentation is heterogenous and may vary from apparently asymptomatic to severe gastrointestinal or extraintestinal symptoms.^2 3^ The only available treatment is a life-long gluten-free diet, which—if adequately maintained—usually results in clinical and histological recovery.^2 4^ However, a substantial part of the patients may suffer from persistent symptoms despite a strict diet,^5–7^ although it may be challenging to distinguish between symptoms caused by coeliac disease and possible coexisting conditions such as irritable bowel syndrome.^8–10^ Particularly long diagnostic delay and severe symptoms at diagnosis have been associated with persistence of symptoms in adult patients with coeliac disease.^5 6 11 12^

Patients diagnosed with coeliac disease in childhood comprise in many ways a special group, as they generally have shorter diagnostic delay, different clinical features, fewer comorbidities and possibly better dietary adherence than subjects diagnosed in adulthood.^13–15^ Diagnosis in childhood may also affect how patients experience coeliac disease, as they have grown up with the disease and may not even remember the times and symptoms before the diagnosis. Persistence of symptoms may undermine patients’ motivation to maintain the burdensome treatment, especially if the importance of and reasons for the diet are not clear. Furthermore, in childhood the gluten-free diet is mainly controlled by the caregivers^15^ and responsibility for dietary adherence shifts to patients themselves during the transition to adulthood.^16^ The prevalence and associated factors for persistent symptoms in individuals diagnosed already in childhood are currently unclear. Our aim here was to evaluate these aspects in originally paediatric and currently adult patients with coeliac disease after long-term gluten-free diet.

## Methods

### Patients and study design

The study was carried out at Tampere University and Tampere University Hospital. A diagnosis search of the patient records of Tampere University Hospital and ascertaining participation in an earlier coeliac disease study revealed that altogether 955 patients had been diagnosed with coeliac disease under the age of 18 years in the period 1966–2014.^17^ Their comprehensive medical data were collected from patient records. Altogether 559 out of the 955 patients were currently adults (≥18 years) and were invited to complete structured study questionnaires and 239 (42%) responded. The responders were more often women (69% vs 52%, p<0.001) and relatives of patients with coeliac disease (56% vs 44%, p=0.035) and suffered less often from type 1 diabetes (9% vs 16%, p=0.029) than the non-responders whereas the groups did not differ in age, clinical features or severity of the duodenal lesion at coeliac disease diagnosis or in the presence of short-term follow-up in childhood.^17^ Based on our structured study questionnaire, altogether 59 (25%) subjects reported occasional or more frequent gluten use and were thus defined not adherent to the diet and excluded from the study. The final study population thus consisted of 180 strictly adherent adult patients with coeliac disease diagnosed in childhood. They were divided into two subgroups according to presence or lack of self-reported persistent symptoms defined as any ongoing gastrointestinal or extraintestinal complaints considered to be related to coeliac disease.

### Characteristics at childhood coeliac disease diagnosis

The diagnostic data collected included the main reason for coeliac disease suspicion, presence and severity of clinical symptoms and the degree of small-bowel mucosal atrophy. In addition, the levels of blood haemoglobin and serum coeliac disease-related autoantibodies were recorded as available.

The reason for coeliac disease suspicion was classified as (1) gastrointestinal symptoms (eg, abdominal pain, constipation, diarrhoea and vomiting), (2) extra-intestinal complaints (eg, dermatological symptoms, arthralgia, anaemia and poor growth) or (3) at-risk group screening. Severity of symptoms was further categorised according to their frequency and impact on daily life as (1) none, (2) mild or (3) moderate/severe. Growth was evaluated according to Finnish growth charts.^18^

All coeliac disease diagnoses were based on duodenal biopsy as recommended by the national guidelines during the study period. The degree of the diagnostic lesion was further graded to (1) partial, (2) subtotal or (3) total villous atrophy. This grading has been used in our clinical practice systematically since the 1960s and corresponds approximately to Marsh-Oberhuber classes IIIa–c.^19^

Anaemia was defined as a haemoglobin value below age-dependent and sex-dependent reference.^20^ Serum IgA class antireticulin and endomysium antibodies, which closely resemble each other, have been measured in our clinical practice since the early 1980s with indirect immunofluorescense.^21^ The results are reported as negative or positive (titre 1:≥5), the latter being further diluted up to 1:4000. Serum IgA class tissue transglutaminase antibodies have been measured since the early 2000s by enzyme-linked immunosorbent assay (Phadia AB, Uppsala, Sweden) and later by an automated recombinant based enzyme fluoroimmunoassay (Phadia). Values ≥7 U/L are considered positive.

### Follow-up in childhood

Presence of short-term follow-up in childhood was evaluated from the medical records 6–24 months after the coeliac disease diagnosis. Treatment response was defined as alleviation of possible symptoms and decrease/normalisation of the serum coeliac antibody levels. Adherence to a gluten-free diet was classified as (1) strict or (2) lapsing according to evaluation by the physician in charge and self-reporting by the patients/caregivers.

### Health outcomes in adulthood

Structured questionnaires were used to elicit adherence to gluten-free diet and presence of possible daily life restrictions due to the diet (eg, when eating at a restaurant, travelling or visiting friends), as well as the presence and nature of possible persistent symptoms. In addition, the participants reported their employment and studying status, current self-perceived health and possible health concerns, presence of offspring, regular physical exercise, previous or current smoking, comorbidities, coeliac disease in the family, membership of a coeliac society and follow-up for coeliac disease. Self-perceived health was classified as (1) excellent, (2) good or (3) moderate/poor; health concerns as (1) none, (2) minor or (3) moderate/severe; and follow-up as (1) regular, (2) sporadic or (3) no follow-up. Current weight and height were elicited to calculate body mass index (BMI, kg/m^2^).

Self-perceived gastrointestinal symptoms and health-related quality of life were evaluated in more detail using the Gastrointestinal Symptoms Rating Scale (GSRS)^22^ and the Psychological General Well-Being Questionnaires (PGWB),^23^ respectively. The GSRS consists of 15 questions scored with a 7-point Likert scale where higher scores indicate more severe symptoms. Total score is calculated as a mean of all questions, and subdimensions for diarrhoea, indigestion, constipation, abdominal pain and reflux as means of the related questions.^22^ The PGWB comprises 22 questions rated from 1 to 6, higher sum of scores representing better well-being.^23^ Subdimensions for anxiety, depression, positive well-being, self-control, general health and vitality are calculated as sum of scores of selected questions.^23^

### Statistics

Numerical variables are reported as medians with quartiles and categorised data as percentages. Comparisons between the two unpaired study groups were made with Mann-Whitney U test in numerical variables as these were non-normally distributed and with χ^2^-test or Fisher’s exact test in categorised variables, depending on the sample size. Difference between the study groups in the prevalence of other gastrointestinal diseases was adjusted with logistic regression analysis. A p<0.05 was considered significant. SPSS V.26 (IBM Corporation) was used for the statistical analyses.

## Results

Altogether 33 (18%) of the 180 currently adult participants reported persistent symptoms despite a strict gluten-free diet and 55% had ≥2 separate symptoms. The most commonly reported symptoms were gastrointestinal (73%), other frequent symptoms being arthralgia (39%), fatigue (39%), skin symptoms (12%) and depression (6%).

The currently symptomatic and asymptomatic patients did not differ significantly at childhood diagnosis in age, sex, year of diagnosis, laboratory values, clinical presentation, symptoms or degree of villous atrophy (table 1). In addition, the groups had comparable short-term dietary adherence (strict diet 86% vs 88%, p=0.797), treatment response (100% vs 99%, p=1.000) and presence of follow-up (87% vs 95%, p=0.097) 6–24 months after diagnosis.

**Table 1:** Clinical characteristics at the time of childhood coeliac disease diagnosis in 180 currently adult patients on a strict gluten-free diet with and without persistent symptoms

	Persistent symptoms in adulthood				P value
	Yes, n=33		No, n=147		
	Median	Q_1_, Q_3_	Median	Q_1_, Q_3_	
Age, years	10.1	4.8, 14.2	9.5	5.8, 13.6	0.716
Year of diagnosis	1995	1982, 2004	1999	1987, 2004	0.270
Haemoglobin, g/L	123	117, 131	124	113, 132	0.677
EmA/ARA§, titre	1:500	1:100, 1:2000	1:200	1:100, 1:1000	0.131
TGA, U/mL	86	26, 120	76	33, 120	0.876
	**n**	**%**	**n**	**%**	
Girls	24	73	101	69	0.651
Main clinical presentation					0.905
*Screen-detected*	6	18	28	19	
*Gastrointestinal*	19	58	78	53	
*Extra-intestinal*	8	24	40	27	
Severity of symptoms‡					0.851
*None*	8	30	33	30	
*Mild*	11	41	39	35	
*Moderate or severe*	8	30	38	35	
Specific symptoms					
*Abdominal pain*	10	37	59	48	0.302
*Anaemia*	13	41	40	29	0.200
Arthralgia*	1	5	6	7	1.000
*Constipation*	3	10	11	9	0.733
*Diarrhoea*	14	47	58	46	0.950
*Poor growth*	15	47	62	47	0.992
*Skin symptoms†*	2	10	8	8	0.677
*Vomiting*	2	7	13	10	0.737
Degree of villous atrophy					0.330
*Partial*	8	30	39	29	
*Subtotal*	8	30	57	43	
*Total*	11	41	37	28	
Type 1 diabetes	0	0	8	7	0.356

*Data available for >80% of patients except in: 112.

†Data available for >80% of patients except in: 124.

‡Data available for >80% of patients except in: 137.

§Data available for >80% of patients except in: 130.

ARA, serum antireticulin antibodies; EmA, serum endomysium antibodies; Q_1_ and Q_3_, lower and upper quartiles; TGA, tissue transglutaminase antibodies.

In the current evaluation a median of 18 years after the diagnosis, patients with persistent symptoms had coexisting gastrointestinal conditions more often than those without symptoms (table 2). The groups did not differ in other comorbidities, sociodemographic characteristics, BMI, family history of coeliac disease, health behaviour or presence of regular follow-up (table 2).

**Table 2:** Current sociodemographic and clinical characteristics of 180 adult patients with childhood coeliac disease diagnosis with and without self-reported persistent symptoms while on a strict gluten-free diet

	Persistent symptoms in adulthood				P value
	Yes, n=33		No, n=147		
	Median	Q_1_, Q_3_	Median	Q_1_, Q_3_	
Age, years	31.6	21.1, 45.5	26.5	22.1, 35.8	0.417
Time from diagnosis, years	20.9	12.5, 34.3	16.8	12.2, 27.0	0.250
Body mass index, kg/m^2^	24.4	22.0, 26.6	22.9	21.2, 26.3	0.210
	**n**	**%**	**n**	**%**	
Sociodemographic characteristics					
Working full-time or part-time*	20	83	88	77	0.507
*Student*	13	39	50	34	0.558
*Offspring*	17	52	60	41	0.275
*Coeliac disease in the family*	21	66	90	62	0.707
Health behaviour					
*Member of coeliac society*	18	55	79	55	0.995
*Current or previous smoking*	12	36	40	27	0.306
*Regular physical exercise*	27	84	133	92	0.198
*Use of gluten-free oats*	29	88	138	95	0.124
*Follow-up of coeliac disease*	6	18	43	29	0.197
Comorbidities					
*Allergy†*	13	41	60	41	0.937
*Asthma*	3	9	16	11	1.000
*Autoimmune thyroidal disease*	3	9	12	8	1.000
*Depression*	4	12	16	11	1.000
*Gastrointestinal disease‡*	6	19	8	6	**0.023**
*Malignancy§*	0	0	3	2	1.000
*Osteoporosis/osteopenia*	0	0	4	3	1.000
*Rheumatic disease*	1	3	7	5	1.000
*Skin disease¶*	5	16	23	16	0.993
*Type I diabetes*	1	3	8	6	1.000

*Data available for >95% of patients except in 138.

†For example, for pollen, animals, antibiotics, food.

‡For example, peptic ulcer, inflammatory bowel disease, irritable bowel syndrome, gastritis.

§For example, breast cancer and non-Hodgkins lymphoma.

¶For example, atopic dermatitis.

Q1 and Q3, lower and upper quartiles.

**Figure 1:**
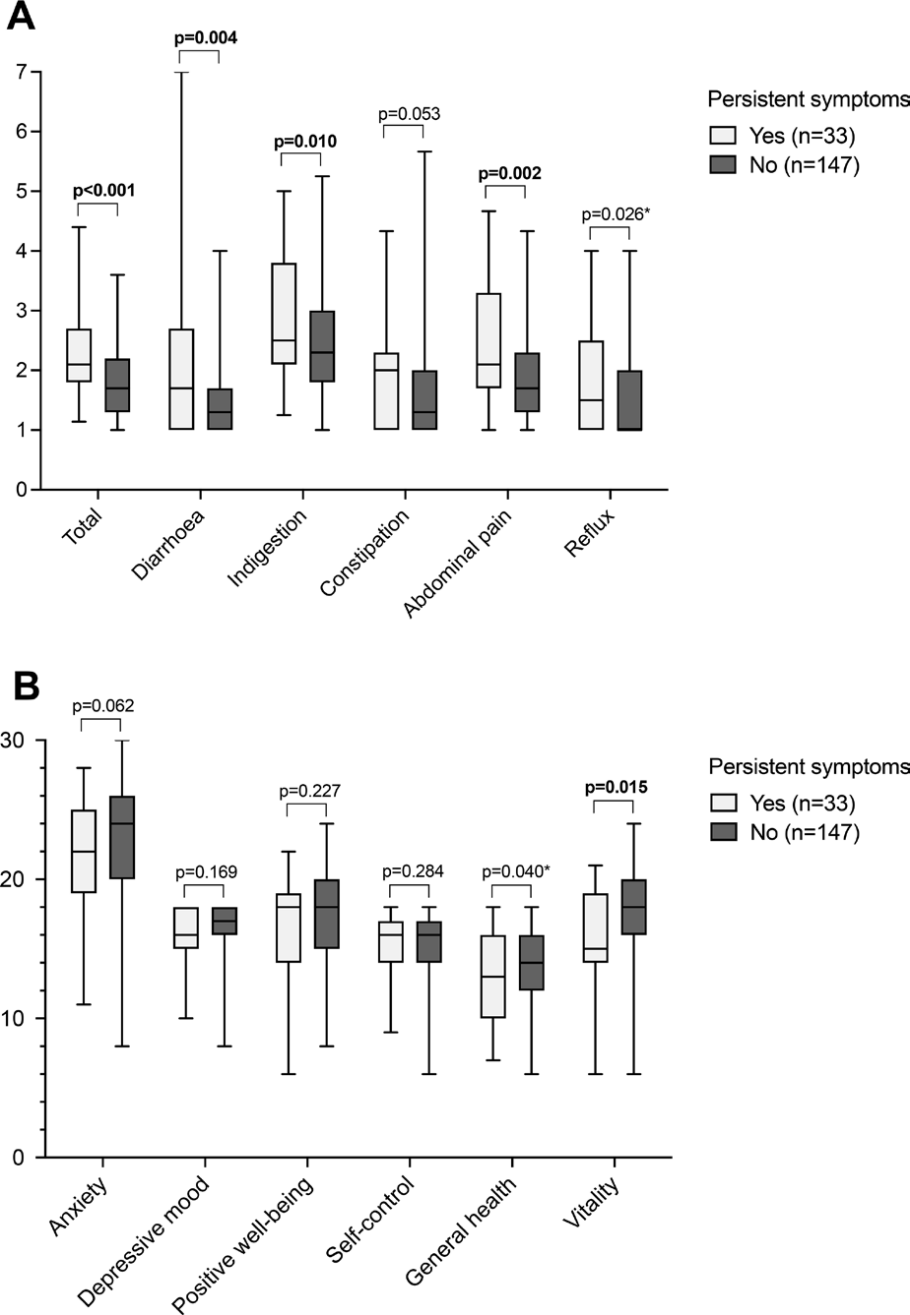
Gastrointestinal symptoms (A) and health-related quality of life (B) in 180 patients diagnosed in childhood with coeliac disease and reporting strict adherence to gluten-free diet in adulthood after a median of 18 years. Patients with and without persistent symptoms are compared and median (horizontal line), lower and upper quartiles (box) and range (vertical line) are shown for both groups. Higher scores denote more severe symptoms or better quality of life. P value marked with an asterisk was no longer significant after adjusting for other concomitant gastrointestinal diseases.

The current GSRS total, diarrhoea, indigestion, abdominal pain and reflux scores were higher, indicating more severe symptoms in patients reporting persistent symptoms, whereas there was no significant difference in constipation (figure 1A). Excluding reflux (p=0.123), the results remained similar after adjusting for presence of gastrointestinal comorbidites.

**Figure 2:**
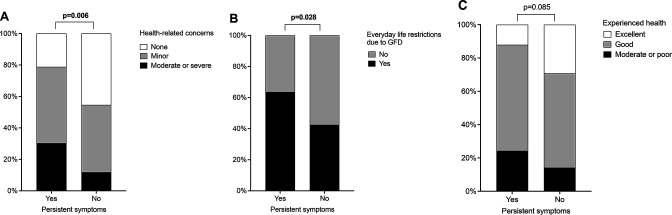
Health-related concerns (A), everyday life restrictions due to gluten-free diet (B) and overall health (C) in 180 patients diagnosed in childhood with coeliac disease and reporting strict adherence to a gluten-free diet in adulthood after a median of 18 years. Patients with and without persistent symptoms are compared. GFD, gluten-free diet.

Patients with persistent symptoms reported poorer general health and vitality scores on the PGWB, while other aspects of quality of life were comparable (figure 1B). After adjusting for gastrointestinal comorbidities, the difference in general health was no longer significant (p=0.075). Health-related concerns (figure 2A) and experience of everyday life restrictions due to gluten-free diet (figure 2B) were more common among those with persistent symptoms, but there was no difference in self-experienced overall health (figure 2C). The results did not change significantly after adjusting for gastrointestinal comorbidities.

## Discussion

We found that 18% of patients diagnosed with coeliac disease in childhood reported persistent symptoms despite a strict gluten-free diet in adulthood. Although the majority of symptoms were gastrointestinal, patients also reported diverse extraintestinal complaints. For comparison, Sciepatti *et al* recently reported that persistent symptoms despite good self-reported dietary adherence affected 38% of originally paediatric patients at their first adult consultation.^7^ On a short-term gluten-free diet, symptoms have been reported in 34% of paediatric^6^ and in 36%–52% of adult patients with coeliac disease.^5 6^ Although these figures overlap, Sansotta *et al* reported symptom resolution to be better in children than in adults.^6^ Most earlier studies have focused solely on gastrointestinal symptoms^5 10 24^ or have not described the nature of symptoms in detail,^7^ whereas Sansotta *et al* described the improvement of various extra-intestinal complaints to be poorer than gastrointestinal symptoms in both children and adults.^6^ Taken together, our patients diagnosed in childhood reported fewer persistent symptoms on a strict diet in adulthood compared with the previously published figures. This could be explained by the longer time elapsing from the diagnosis and differing definitions of a strict diet as the most common reason for the symptoms is gluten exposure. Also, early diagnosis in childhood may decrease the risk for persistent symptoms compared with diagnosis in adulthood.

Persistent symptoms in adulthood could not be predicted at childhood coeliac disease diagnosis or during short-term follow-up. However, currently symptomatic patients reported an overrepresentation of gastrointestinal comorbidities. The existing data on a possible association between persistent symptoms and presence of comorbidities are somewhat inconsistent.^6 10–12 24 25^ This may be due to differences in the age, background and overall health of the study patients which likely affect the development of comorbidities. For example, an important but rare entity, refractory coeliac disease has not been reported in paediatric patients but should be ruled out especially in those with long diagnostic delay, older age and poor treatment response.^26^ Also, the definition for comorbidities included in the analyses may explain some of the differences between the studies. Of note, persisting symptoms may fullfill the diagnostic criteria of functional gastrointestinal disorders as such and thus be counted as comorbidities.^10 24 27^ Sainsbury *et al* reported in their meta-analysis functional gastrointestinal disorders to affect 29% of patients with coeliac disease compared with 10% of controls.^28^ Conversely, when patients with insufficient response to gluten-free diet are evaluated further, other comorbidities contributing to the symptoms can often be found.^29 30^ Furthermore, factors associated especially with the persistent symptoms in coeliac disease and other gastrointestinal disorders, including changes in the gut microbiota, small-intestinal bacterial overgrowth and low-level mucosal inflammation may overlap, although more data about the pathogenesis, diagnostics and prevalence figures are needed.^31–35^

Regardless of the cause, it is important to identify ongoing symptoms, particularly as we detected them to be associated with everyday life restrictions and health concerns and impaired quality of life. Consistent with this, we and others have previously observed an association between symptom persistency and poorer quality of life among treated patients with coeliac disease.^11 12 25 36^ However, in most of these studies the possible concomitant effects of dietary lapses were not noted.^11 12 25^ Challenges in maintaining a strict gluten-free diet may also predispose patients to anxiety and poor quality of life, making it difficult to distinguish between the effects of dietary restriction and ongoing symptoms.^37 38^ In any case, the negative effects on daily life of persistent symptoms serve to stress the importance of individualised follow-up and support for patients with poor treatment response. In these circumstances, the transition phase from paediatric to adult care may be particularly important as this is the period when the basis for good long-term treatment and health outcomes is built.

The main strengths of our study were the large and representative group of originally paediatric patients with coeliac disease, carefully verified diagnoses and particularly long follow-up time. Limitations included retrospective data collection at diagnosis and short-term follow-up in childhood, and only moderate survey response rate. Selection bias regarding those responding the questionnaires could affect the prevalence figures of persistent symptoms as well as other patient characteristics, although responders and non-responders did not differ in most variables at childhood diagnosis or follow-up. Another limitation was that the definition of persistent symptoms was based on self-reporting. On the other hand, this enabled us to include those suffering from persistent extra-intestinal complaints. Finally, adherence to a gluten-free diet was self-reported instead of using validated questionnaire, serology or determination of gluten immunogenic peptides, and some patients could have been exposed to gluten inadvertently.^39 40^ However, also these potentially more objective methods to evaluate the adherence have their limitations.^41 42^

To conclude, we found persistent symptoms despite a strict gluten-free diet to be common after transition to adulthood. Besides characteristic gastrointestinal symptoms, the patients also reported a variety of extraintestinal complaints. These may impair quality of life and cause unnecessary worries and burden and are thus important to recognise. Moreover, evaluations for possible contributory, especially gastrointestinal comorbidities are justified in those reporting persistent symptoms despite successful treatment.

## Data Availability

All data relevant to the study are included in the article or uploaded as supplementary information.
